# Acenocoumarol Pharmacogenetic Dosing Algorithm versus Usual Care in Patients with Venous Thromboembolism: A Randomised Clinical Trial

**DOI:** 10.3390/jcm10132949

**Published:** 2021-06-30

**Authors:** Hoi Yan Tong, Alberto M. Borobia, Manuel Quintana-Díaz, Sara Fabra, Manuel González-Viñolis, Carmen Fernández-Capitán, María A. Rodriguez-Dávila, Alicia Lorenzo, Ana María López-Parra, Nuria Ruiz-Giménez, Francisco Abad-Santos, Carmen Suarez, Olga Madridano, Jorge Francisco Gómez-Cerezo, Pilar Llamas, Carlos Baeza-Richer, Eduardo Arroyo-Pardo, Antonio J. Carcas, The PGX-ACE Spanish Investigators Group

**Affiliations:** 1 Clinical Pharmacology Department, La Paz University Hospital, IdiPAZ, 28046 Madrid, Spain; dochoiy29@gmail.com; 2 Pharmacology Department, School of Medicine, Autonomous University of Madrid, IdiPAZ, 28046 Madrid, Spain; 3 General Emergency Department, La Paz University Hospital, IdiPAZ, 28046 Madrid, Spain; mquintanadiaz@gmail.com (M.Q.-D.); sarafabra@yahoo.com (S.F.); mglezvinolis@gmail.com (M.G.-V.); 4 Internal Medicine Department, La Paz University Hospital, IdiPAZ, 28046 Madrid, Spain; mfcapitan@salud.madrid.org (C.F.-C.); marodavila@ono.com (M.A.R.-D.); alicia.loher@gmail.com (A.L.); 5 Laboratory of Population and Forensic Genetics, Department of Legal Medicine, Psyquiatry and Patology, Faculty of Medicine, Complutense University of Madrid, 28040 Madrid, Spain; amlopez236@gmail.com (A.M.L.-P.); cbaezaricher@med.ucm.es (C.B.-R.); earroyop@med.ucm.es (E.A.-P.); 6 Department of Internal Medicine, University Hospital La Princesa, 28006 Madrid, Spain; airunruizg@hotmail.com (N.R.-G.); mcarmen.suarez@uam.es (C.S.); 7 Clinical Pharmacology Department, University Hospital La Princesa, Teófilo Hernando Institute, Autonomous University of Madrid (UAM), Health Research Institute La Princesa (IIS-IP), 28006 Madrid, Spain; francisco.abad@uam.es; 8 Department of Internal Medicine, University Hospital Infanta Sofía, 28702 Madrid, Spain; olgamadridano@hotmail.com (O.M.); jfrancisco.gomez@salud.madrid.org (J.F.G.-C.); 9 Department of Internal Medicine, University Hospital Jiménez Díaz Foundation, 28040 Madrid, Spain; pilar.llamas@uam.es

**Keywords:** pharmacogenetics, acenocoumarol, venous thromboembolism, clinical trial

## Abstract

Patients with venous thromboembolism (VTE) require immediate treatment with anticoagulants such as acenocoumarol. This multicentre randomised clinical trial evaluated the effectiveness of a dosing pharmacogenetic algorithm versus a standard-of-care dose adjustment at the beginning of acenocoumarol treatment. We included 144 patients with VTE. On the day of recruitment, a blood sample was obtained for genotyping (CYP2C9*2, CYP2C9*3, *VKORC1*, *CYP4F2*, *APOE*). Dose adjustment was performed on day 3 or 4 after the start of treatment according to the assigned group and the follow-up was at 12 weeks. The principal variable was the percentage of patients with an international normalised ratio (INR) within the therapeutic range on day 7. Thirty-four (47.2%) patients had an INR within the therapeutic range at day 7 after the start of treatment in the genotype-guided group compared with 14 (21.9%) in the control group (*p* = 0.0023). There were no significant differences in the time to achieve a stable INR, the number of INRs within the range in the first 6 weeks and at the end of study. Our results suggest the use of a pharmacogenetic algorithm for patients with VTE could be useful in achieving target INR control in the first days of treatment.

## 1. Introduction

Over the years, vitamin K antagonists (VKAs) have shown high efficiency in preventing thromboembolic complications, mainly in patients with atrial fibrillation and cardiac valve replacement; however, VKAs are also employed for the acute-phase treatment of patients with venous thromboembolism (VTE), both in pulmonary thromboembolism (PTE) and in deep vein thrombosis (DVT) [1,2].

VKAs such as warfarin and acenocoumarol are characterised by their narrow therapeutic range and high inter-individual variability, which make it difficult for them to achieve and maintain international normalised ratio (INR) values within the recommended target range. Numerous studies have been conducted to find factors that could explain this variability and have shown that certain demographic data and genetic information, mainly allelic variants of *CYP2C9* (rs1799853 and rs1057910) and *VKORC1* (rs9923231), can affect the dose required to achieve a stable INR within the therapeutic range for warfarin and acenocoumarol, and *CYP4F2* (rs2108622) can affect the dose required for acenocoumarol. Due to the evidence from these studies, the US Food and Drug Administration updated the warfarin drug label information on dosing recommendations in 2007, with consideration for pharmacogenetic polymorphism information [3]. Among the coumarins, warfarin is the most widely known VKA and is employed in numerous countries; acenocoumarol, however, is the main coumarin employed in many European countries. The drug labels for acenocoumarol in Europe only provide general pharmacogenetic information but no specific recommendations for dosing.

Several algorithms have been designed to individualise warfarin and acenocoumarol doses, taking into account demographic, clinical and pharmacogenetic factors. In 2005, Sconce et al. published the first warfarin dosing algorithm, which included age, height and *CYP2C9* and *VKORC1* genotype, an algorithm that could explain nearly 55% of the variability in the drug dosage [4]. Since then, other algorithms have been published [5,6,7], with a number of algorithms for acenocoumarol, which can predict 40–60% of the variability in the dosage of this drug [8,9,10,11,12,13,14].

Clinical trials to prove the utility of the pharmacogenetics in this setting were conducted after the algorithms were developed, with several trials on warfarin, comparing genotype-guided dosing versus conventional dosing and versus a clinical algorithm; however, the results have been inconsistent [15,16,17,18,19,20,21,22,23]. A number of meta-analyses have been published showing that genotype-guided dosing improves the percentage of time within the therapeutic INR range [24,25,26], although the usefulness of the pharmacogenetics in reducing bleeding complications is a subject of debate [24,26,27,28]. To date, there have been only two published clinical trials on acenocoumarol evaluating two different pharmacogenetic algorithms, with conflicting results [22,23], and a few meta-analyses within the context of studies assessing the effect of including genetic variables in the dosing of coumarins [25,28,29,30]. Most of these studies (with both warfarin and acenocoumarol) included a mixed population. Differentiating patients with atrial fibrillation from those with VTE is important, given that the latter group requires proper anticoagulation very soon after the diagnosis to prevent the onset of possible complications (both early and late), such as re-thrombosis, post-thrombotic syndrome and pulmonary hypertension [31]. Our data [14] show that patients with VTE are younger and take a slightly higher weekly dose than patients with atrial fibrillation. Our research group designed the first and, as of yet, only acenocoumarol algorithm for patients with VTE that included the following clinical variables: age, body mass index, enzyme inducer status, amiodarone status and genetic factors (*CYP2C9*, *VKORC1*, *CYP4F2* and *APOE*). Although clinical variables can explain only 22% of the dose variability [12], this pharmacogenetic algorithm explains 60.6% of the dose variability and has performed well in an external cohort [32]. The algorithm can be used via the following web link: http://www.dosisacenocumarol.com/en/index.php (accessed on 15 March 2021). The variables mentioned above have to be included and the recommended dose to initiate treatment is calculated.

Our next step was to design and implement a clinical trial aimed at evaluating the effectiveness and efficiency of an acenocoumarol pharmacogenetic dosing algorithm compared with the standard-of-care dosage adjustment for patients with VTE. 

## 2. Methods

### 2.1. Trial Design

Between March 2011 and September 2013, we conducted a pragmatic, multicentre, randomised, parallel, 2-arm, single-blind clinical trial, as described previously [33]. The study was approved by the Ethics Committee of La Paz University Hospital and by the Spanish Agency of Medicine and Health Products, registered in EudraCT (number: 2009-016643-18).

### 2.2. Study Population

The target population consisted of patients with newly diagnosed VTE, including PTE and DVT, who were starting therapy with acenocoumarol. The detailed inclusion and exclusion criteria have been previously described [33]. The study was authorised to be conducted in 5 hospitals in the Community of Madrid, Spain; however, one of the hospitals encountered recruitment problems, leaving 4 participating hospitals: La Paz University Hospital, La Princesa University Hospital, Hospital Infanta Sofía and Fundación Jiménez Díaz. Patients were recruited from these centres’ internal medicine and emergency departments, and all participants provided their written informed consent.

### 2.3. Randomisation and Treatment

After inclusion in the trial, the patients were randomly assigned in a 1:1 ratio to either of 2 arms: dose adjustment using the pharmacogenetic algorithm (PGx) or standard adjustment (control). The randomisation was performed using a masked randomisation scheme, in blocks of 4 patients and stratified by centre, using a Microsoft Excel program. Randomisation envelopes containing the assigned strategy were created for each centre with the patient’s code written on the outside of these envelopes. Both the envelope and the card were stored in the principal investigator’s file. The subinvestigator responsible for recruitment was in charge of opening the envelopes according to patient code.

Initially, all patients were administered a standard acenocoumarol dose along with low molecular weight heparin (LMWH). On the third or fourth day of treatment (day 0 of the study), the dose adjustment was performed according to the assigned group:

- PGx group: The dose was calculated using the pharmacogenetic algorithm, which included demographic (age, sex, weight and height), clinical (use of amiodarone or inducer drugs) and pharmacogenetic (polymorphisms of *CYP2C9*, *VKORC1*, *APOE* and *CYP4F2*) variables [12].
- Control group: The dose was adjusted according to the standard procedure in routine clinical practice. As a guideline for handling and adjusting the dosages, the researchers employed the local guideline developed for managing patients treated with acenocoumarol.

### 2.4. Study Procedures

The patients who agreed to participate in this clinical trial had to perform a total of 8 visits in a period of 12 weeks (Table 1).

Patients with newly diagnosed VTE were identified from the emergency department or from the hospital ward within the first 48 h of the diagnosis. Those patients who met all of the inclusion and none of the exclusion criteria were asked to participate in the study. Before undergoing any study procedure, we confirmed that the patients had signed the informed consent.

On the enrolment day (days −3 or −2), all patients began treatment with acenocoumarol according to the standard clinical practice. The recommended approach was to start with 2 mg a day for patients younger than 65 years and 1 mg for older patients or those with a bleeding risk factor. In all cases, the patients took LMWH, at least until the INR was within the therapeutic range. On the enrolment day, a blood sample (2 ethylenediaminetetraacetic acid tubes of 5 mL) was extracted and sent for genotyping. The pharmacogenetic results were returned within 48 h.

### 2.5. Genotyping

Genotyping was performed by the Laboratory of Population and Forensic Genetics of the Department of Legal Medicine, Psychiatry and Pathology of the Faculty of Medicine at the Complutense University of Madrid, according to a previously described multiplex technique [34]. Genotyping included CYP2C9*2 (C → T = rs1799853), CYP2C9*3 (A → C = rs1057910), *VKORC1* (3588 G → A = rs9923231), *CYP4F2* (23454 G → A = rs2108622) and *APOE* (8041 C → T = rs7412).

On day 0 of the study and thereafter, the INR was determined from a capillary blood sample using a CoaguChek^®^ portable coagulometer (Roche Farma SA, Madrid, Spain), and the dose was adjusted according to the group to which the patient had been randomised. All patients were followed-up for 3 months, with their INR measured on days 3, 7, 15, 30, 60 and 90 of the study. Additional unscheduled visits could be arranged, if necessary.

Adverse events were recorded during each visit through open-ended questions, physical exploration and spontaneous reporting by the patients. We employed Naranjo’s algorithm to evaluate causality [35]. Re-thrombosis and haemorrhagic events were considered adverse events of special interest.

### 2.6. Outcome Variables

The study’s primary endpoint was the percentage of patients with an INR within the therapeutic range (INR between 2 and 3) on day 7 after the start of treatment (visit 3). Co-primary endpoints included: (a) the time from the start of the oral anticoagulant therapy to achieving a stable INR within the therapeutic range (INR within the therapeutic range for 3 consecutive measurements at least 2 weeks apart, with a maximum difference between the daily average doses of 10%); (b) the number of INRs within the range in the first 6 weeks of treatment; and (c) the number of INRs within the range during the 3 months of the study. The secondary endpoints were the proportion of time within the therapeutic range from the start of treatment to the end of the follow-up; the number of additional INR measurements during the study; and the proportion of adverse events until the end of the follow-up.

### 2.7. Statistical Analysis

We calculated the sample size to detect an absolute difference of 20% in the number of patients within the therapeutic range on day 7 (visit 3) after starting the treatment with acenocoumarol. Considering alpha and beta errors of 0.05 and 0.2, respectively, and assuming a 20% rate of patients lost to follow-up, the number of patients needed was 120 per study group.

There were 3 study populations: the intention-to-treat (ITT) population, defined as all randomised participants who met the selection criteria, took the study medication and had at least 1 measurement for the main efficacy variable (INR); the per protocol (PP) population, defined as all randomised participants who met the inclusion criteria, took the study medication, had at least 1 measurement for the main efficacy variable (INR) and did not miss more than 1 visit or the last visit; and the safety population, defined as all randomised participants who took at least 1 treatment dose. The main study analysis was performed using the ITT strategy.

For all primary and secondary efficacy variables related to the number of events (the number of INRs within the therapeutic range during the first 6 weeks of treatment, the number of INRs within the therapeutic range at the end of treatment, the extraordinary determinations of INRs, the number of INRs <1.5 and the number of INRs >4), we employed nonparametric approaches. We compared the median (95% CI) and median differences (95% CI) using Hodges–Lehmann estimates by means of the Mann–Whitney test and Poisson analysis.

We estimated the time to event (INR stabilisation) using the Kaplan–Meier approach and compared the treatments with the stratified log-rank test, employing Cox regression models to estimate the hazard risks and 95% CI. We estimated the proportion of time within the therapeutic range by means of linear interpolation and compared the treatments by means of Student’s t-test.

All statistical tests were applied with a 0.05 2-sided significance level. We tested the Hardy–Weinberg equilibrium to calculate the allele frequencies and performed the safety analysis using the number of adverse events reported during the study, including the number of INRs >4 or <1.5, applying the chi-squared test.

We performed the statistical analysis using the SAS system (Release 9.2).

## 3. Results

### 3.1. Patients

**Figure 1:**
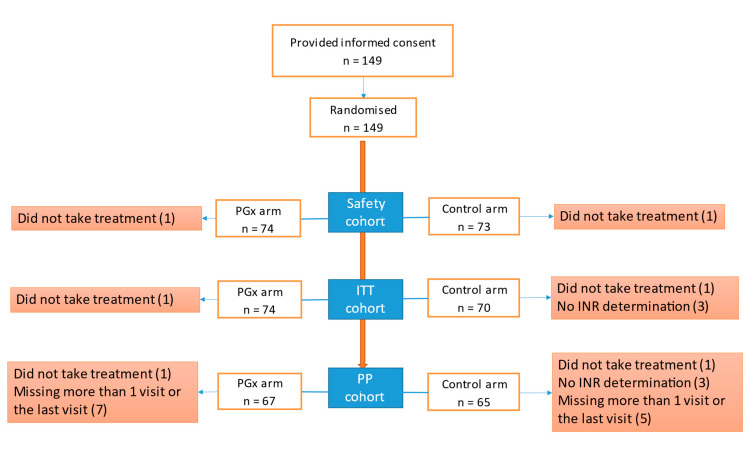
Flow chart of included patients. Abbreviations: ITT, intention-to-treat; PGx, pharmacogenetic algorithm; PP, per protocol.

We recruited 149 patients, 75 of whom were randomised to the PGx group and 74 of whom were randomised to the control group (Figure 1). We did not achieve the calculated sample size due to the low recruitment rate. The ITT population consisted of 144 patients (74 in the PGx arm and 70 in the control arm). All reported efficacy results are from the ITT group, whose mean age was 59.42 (18.53) years. We recruited 77 (53.5%) men and 67 (46.5%) women. The diagnosis was DVT in 61.8% of the patients, PTE in 22.2% and both diagnoses in 16%. Table 2 shows the demographic data. The gene frequencies are presented in a Hardy–Weinberg equilibrium.

### 3.2. Primary Outcomes

#### 3.2.1. INR within Therapeutic Range at Day 7 of Treatment

This was the main primary endpoint of the study. Eight patients did not have a valid INR at day 7; 72 patients in the PGx and 64 in the control group were therefore included in the primary statistical analysis. Thirty-four (47.2%) patients had an INR value within the therapeutic range (2 to 3) at day 7 after the start of treatment in the PGx group compared with 14 (21.9%) patients in the control group, a difference that was statistically significant (*p* = 0.0023). After including the eight patients with no INR values at day 7, assuming the closest INR to that day, the statistically significant difference between the two groups was maintained.

#### 3.2.2. INR Stabilisation

A stable INR as defined in the protocol was reached by 37 patients, 21 (28.4%) of the 74 patients in the PGx group and 16 (22.9%) of the 70 patients in the control group (*p* = 0.45). The median (range) time from the start of the treatment with acenocoumarol to the first day of stable INR in the PGx group was 17 (10–30) days compared with the 15 (10–28) days for the control group (*p* = 0.7929).

#### 3.2.3. Number of INR Readings within the Therapeutic Range

A total of 128 patients reached at least 1 INR reading within the range in the first 6 weeks of treatment, 66 (89.2%) in the PGx group and 62 (88.6%) in the control group (*p* = 1.000). During the 3 months of treatment, 135 (93.8%) patients had at least 1 INR reading within the therapeutic range, 70 (94.6%) in the PGx group and 65 (92.9%) in the control group (*p* = 0.7402).

### 3.3. Secondary Outcomes

The secondary outcomes did not differ significantly between the two groups. In the global assessment, the PGx group had a mean percentage of time within the therapeutic range of 54.69% (32.06) compared with 58.54% (31.50) of the control group (*p* = 0.4683).

Most of the patients had at least 1 unscheduled INR reading (those visits in addition to the 8 visits established in the study protocol), 55 (74.3%) in the PGx group and 56 (80%) in the control group. Nineteen (25.7%) patients in the PGx group did not have unscheduled visits compared with 14 (20%) in the control group (*p* = 0.4180).

**Figure 2:**
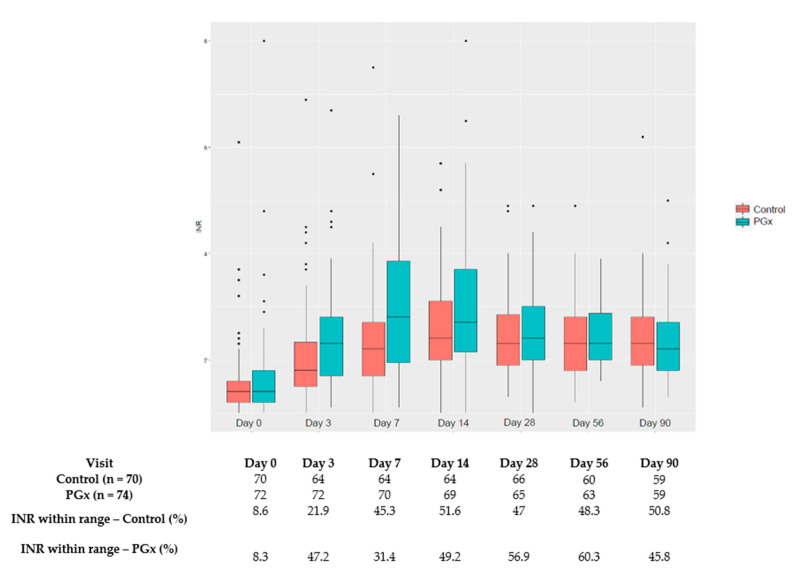
Percentage of patients with INR within therapeutic range per scheduled visit. * outlayers.

The percentage of patients with INR within the therapeutic range per visit is shown in Figure 2.

### 3.4. Safety Outcomes

The incidence of adverse events did not differ significantly between the two groups (Table 3). There were two thromboembolic events, one PTE in the control group and one DVT in the PGx group. There were 6 haemorrhagic events in the PGx group and 11 in the control group. There were no differences in the number of patients with at least one INR reading below 1.5; however, more patients in the PGx group had at least one INR reading above 4 (Table 3), although this difference did not reach statistical significance (*p* = 0.06).

## 4. Discussion

For many years, coumarin drugs were the main anticoagulants on the market worldwide. In recent years, there has been a major advance in the development of new anticoagulant drugs (direct acting anticoagulants—DOACs), with an efficacy and safety profile that has led to a decreasing use of coumarin drugs. However, in the case of Spain, and mainly due to the cost of DOACs, they are not as widely prescribed. By 2019, around 70% of the anticoagulated Spanish population was on acenocoumarol, of which more than half had inadequate INR control [36].

In recent years, numerous pharmacogenetic algorithms have been published in an attempt to improve the dose individualisation of warfarin and acenocoumarol. These algorithms can explain between 40% and 60% of the variability in drug dosing to achieve stable INRs but have certain relevant differences, such as differences in the ethnicity of the included patients, as indicated by Ragia et al. [37]. It might be preferable to use specific algorithms for each ethnic group to achieve a better prediction of the acenocoumarol dosage. The algorithms also differ in terms of the included variables (demographic, clinical and pharmacogenetic) and control arms, with some trials including clinical algorithms and others using standard care, which can differ significantly among countries. The follow-up duration also varies among studies, all of which complicates the comparison of the different algorithms.

Despite the contribution of pharmacogenetic algorithms to explain the variability in the dosing of coumarin drugs, doubts remain about their applicability in routine clinical practice. Several clinical trials and metanalyses have been published to date on warfarin but with inconclusive results [25,26,27,28,29,30]. In the case of acenocoumarol, the results of the only two available clinical trials on acenocoumarol are also inconclusive. Verhoef et al. compared a genotype-guided dosing algorithm versus a dosing algorithm based on only clinical factors. The authors recruited patients with atrial fibrillation (83%) or venous thromboembolism (17%) who started treatment with acenocoumarol. Their findings showed that the use of genotype-guided dosing did not improve the time within the therapeutic INR range during the 12 weeks after the start of treatment [22]. The other clinical trial, conducted by Cerezo-Manchado et al. of patients with atrial fibrillation, showed that the percentage of patients reaching a steady dose within 90 days was higher in the pharmacogenetic arm (39% vs. 25%; *p* = 0.038). The authors also found that the pharmacogenetic group had a higher percentage of therapeutic INRs than the standard care group (50% vs. 45%; *p* = 0.046) [23].

The clinical trial conducted by our group compared an algorithm consisting of demographic, clinical and genetic information versus the usual clinical practice algorithm aimed at determining the initial acenocoumarol dosage needed to achieve an INR between 2 and 3 in patients with newly diagnosed DVT and/or PTE.

Our results show that, at day 7 after starting treatment, 47% of the patients in the pharmacogenetic algorithm arm were within the therapeutic range compared with only 22% in the standard clinical practice arm. When designing the clinical trial, we selected this variable as the main primary endpoint because a delay in achieving an adequate INR in thromboembolic disease is related to adverse outcomes [31,38,39]. We therefore consider that the results confirm the utility of this pharmacogenetics algorithm in achieving a therapeutic INR soon after starting acenocoumarol treatment in this population. Verhoef et al. found similar results, showing that the time within the therapeutic range during the first 4 weeks after starting treatment was longer for the genotype-guided group (52.8% vs. 47.5%). As the authors discussed in their article, however, their analysis was not corrected for multiple testing, and it is possible that this difference was a chance finding [22]. The objective of maximising the time within the therapeutic range is important not only for patients with VTE but also for those with other diseases, as stated by Gage et al. who, in the Genetic Informatics Trial, showed the effectiveness of a pharmacogenetic algorithm in reducing adverse events in the acute setting of patients with elective knee or hip arthroplasty [40].

Two important aspects need to be considered when interpreting these results: The target patients in our trial differed from those of other studies on acenocoumarol that included mainly patients with atrial fibrillation (AF); and our algorithm was developed for patients with VTE, and therefore the target population in this trial should be maintained to validate the algorithm. It is important to emphasise that patients with VTE differ from patients with AF. According to our data, patients with VTE are younger (66 vs. 72.4 years) and require higher weekly doses of acenocoumarol (15 vs. 13.5) than patients with AF [14]. Achieving an INR within the target range as soon as possible after the diagnosis is crucial for this population, and our results show that a pharmacogenetic algorithm can help in achieving this objective, with a number needed to treat (NNT) of 4 to obtain an INR within the therapeutic range during the first week. After the first few days, however, changes are mainly driven by the previous INR results and other factors that influence INR variability in addition to pharmacogenetics. No statistical differences were found in the subsequent INR checks, which could be related to the adjustments made through the usual INR determinations, which was performed for both groups, thereby reducing the positive influence of the initial prediction reached through the pharmacogenetic algorithm. In addition to the well-known clinical practice of adjusting the acenocoumarol dosage, this influence can also be inferred from Figure 2, where the first INR readings are ameliorated in the subsequent INR determinations. This phenomenon has already been observed in a previously published trial [22]. Pharmacogenetic testing would also be especially relevant for those patients requiring extreme doses, given that their dose adjustments through INR monitoring are performed quite slowly, with progressive changes until the required stable dose is reached. According to our data, 51% of the patients required >21 mg/week or ≤11 mg/week. Preemptive genotyping of the patients could improve the results of the pharmacogenetic algorithms. The main limitations of this clinical trial are the following: the use of an intermediate variable, due to the fact that aiming for hard clinical endpoints would require a very large number of patients; the calculated sample size could not be achieved, although we did find a significant difference in the main variable despite this limitation; however, it is uncertain what the outcome would have been for some of the variables assessed that currently have a borderline statistical significance value, such as the safety assessment where possibly more patients in the PGx group would have had an INR value higher than 4. Furthermore, the non-blinded design probably allowed for closer monitoring and acenocoumarol dose adjustments after day 7 and contribute to blur the between groups differences in INR control.

In conclusion, our results suggest that the use of an acenocoumarol pharmacogenetic algorithm for patients with VTE could be useful in achieving better INR control in the first days of treatment, a highly relevant issue in VTE.

## Figures and Tables

**Table 1:** Study procedures.

Visit	1	2	3	4	5	6	7	8
Day of study	−3 or −2	0	3	7	15	30	60	90
Window (day)	0	0	0	+/−1	+/−3	+/−5	+/−7	+/−7
Recruitment	√							
Informed consent	√							
Selection criteria	√							
Medical history	√							
Physical examination	√							√
Blood samples for pharmacogenetics	√							
Randomisation	√							
Acenocoumarol dosing initiation	√							
Acenocoumarol dosage adjustment		√	√	√	√	√	√	√
INR determination		√	√	√	√	√	√	√
Adverse effects evaluation		√	√	√	√	√	√	√

Abbreviation: INR, international normalised ratio.

**Table 2:** Baseline data.

Parameter	PGx Group (*n* = 74)	Control Group (*n* = 70)	Total (*n* = 144)
Age, years	60.12 (19.4)	58.67 (17.7)	59.42 (18.5)
Male sex, n (%)	44 (59.5)	33 (47.1)	77 (53.5)
Race, n (%)			
White/Asian	73 (98.6)/1 (1.4)	70 (100)/0 (0)	143 (99.3)/1 (0.7)
Hospitalised, n (%)	31 (41.9)	32 (45.7)	63 (43.8)
Length of hospitalisation, days	6.33 (4.15)	6.16 (3.03)	6.25 (3.59)
PTE, n (%)	18 (24.3)	14 (20.0)	32 (22.2)
DVT, n (%)	48 (64.9)	41 (58.6)	89 (61.8)
Both PTE and DVT, n (%)	8 (10.8)	15 (21.3)	23 (16)
Height, cm	165.77 (11)	165.99 (11.1)	165.92 (11.1)
Weight, kg	78.44 (16.4)	78.59 (16.4)	78.57 (16.4)
BMI	28.55 (5.3)	28.53 (5.3)	28.55 (5.2)
Amiodarone use	1 (1.4)	0 (0)	1 (0.7)
Inducer use	2 (2.7)	0 (0)	2 (1.4)
CYP2C9 genotype, n (%)			
*1*1	52 (70.3)	43 (61.4)	95 (66.0)
*1*2	14 (18.9)	14 (20.0)	28 (19.4)
*1*3	6 (8.1)	5 (7.1)	11 (7.6)
*2*2	1 (1.4)	5 (7.1)	6 (4.2)
*2*3	1 (1.4)	2 (2.9)	3 (2.1)
*3*3	0	1 (1.4)	1 (0.7)
VKORC1 genotype, n (%)			
GG	31 (41.9)	24 (34.3)	55 (38.2)
GA	31 (41.9)	40 (57.1)	71 (49.3)
AA	12 (16.2)	6 (8.6)	18 (12.5)
CYP4F2 genotype, n (%)			
CC	27 (36.5)	20 (28.6)	47 (32.6)
CT	37 (50.0)	39 (55.7)	76 (52.8)
TT	10 (13.5)	11 (15.7)	21 (14.6)
APOE176 genotype, n (%)			
CC	61 (82.4)	64 (91.4)	125 (86.8)
CT	13 (17.6)	5 (7.1)	18 (12.5)
TT	0	1 (1.4)	1 (0.7)

Values are listed as mean ± SD unless otherwise noted. * Chi-squared test. Abbreviations: BMI, body mass index; DVT, deep vein thrombosis; PGx, pharmacogenetic algorithm; PTE, pulmonary thromboembolism.

**Table 3:** Safety analysis.

AE	PGx Group *n* (%) (*n* = 74)	Control Group *n* (%) (*n* = 73)	Total *n* (%) (*n* = 147)	*p*
Patients with at least 1 AE	10 (13.5)	14 (19.2)	24 (16.3)	0.35
Haemorrhagic events	6 (8.1)	11 (15.1)	17 (11.6)	0.18
Thromboembolic events	1 (1.4)	1 (1.4)	2 (1.4)	0.99
Patients with at least 1 INR reading <1.5	45 (60.8)	50 (68.5)	95 (64.6)	0.18
Patients with at least 1 INR reading >4	31 (41.9)	19 (26)	50 (34)	0.06

Abbreviations: AE, adverse event; PGx, pharmacogenetic algorithm.

## Appendix A

PGx-ACE Investigation Group: Nicolas Medrano-Casique1, Claudia Zegarra^1^, Marta Velasco^1^, Mario Muñoz^1^, Rafael Hernández^1^, Elena Ramírez^1^, Jesús Frías^1^, Giorgina Sal-gueiro^2^, María V. Cuesta-García^3^, M. Soledad García-Muñoz^3^, María Teresa Gómez Rodríguez^4^, Pilar Pérez Egea^4^, Carlos Casanova García^4^, Laura García Regaño^4^, María Azucena Saez Berlana^4^, María Alejandra Rabanal Carrera^4^, María Pilar Martin Cerrato^4^, Manuel Sánchez López^4^, Pablo Astorga Díaz^4^, Milagros Velázquez García^4^, Francisco Javier De La Casa Sánchez^4^, Ángeles Conde Llorente^4^, María Dolores Parejo De Pablos^4^, María Patrocinio Verde González^4^, María Rosa Del Álamo Gutiérrez^4^, Ángeles Brieva Gar-cía^4^, Margarita Encinas Sotillo^4^, Esther Frías Díaz^4^, Francisca Dacal Cubillo^4^, Inés Casas Jiménez^4^, Isabel Sola Vergara^4^, Fernando López Beltrán De Lis^4^, Antonio Vázquez Gallego^4^, María Teresa García Argudo^4^, María Ángeles Bueno Martin^4^, Encarnación Vega Arija^4^, Carmen Belinchón Moya^4^, Gloria Menal Arriazu^4^, María Teresa Gómez Martínez^4^, Ana Román Ruiz^4^, Laura Martin Saez^4^.

1. Department of Clinical Pharmacology. La Paz University Hospital, IdiPAZ, Madrid, Spain
2. Department of Internal Medicine. La Paz University Hospital, IdiPAZ, Madrid, Spain
3. Department of Hematology. La Paz University Hospital, Madrid, Spain
4. Primary Health Care Center “Barrio del Pilar”, Madrid, Spain
